# The impact of *S. cerevisiae* strains on the aroma profile and physicochemical properties of *Phyllanthus emblica* L. fruit wine

**DOI:** 10.1016/j.fochx.2025.103388

**Published:** 2025-12-14

**Authors:** Haibin Yuan, Yanlin Li, Jianhang Tu, Tianyang Wang, Kaixian Zhu, Hongfeng Jia, Huachang Wu, Ping Dong

**Affiliations:** a Cuisine Science Key Laboratory of Sichuan Province, Sichuan Tourism University, Chengdu 610100, China; b College of Culinary and Food Science Engineering, Sichuan Tourism University, Chengdu 610100, China; c Faculty of Food and Biological Engineering, Chengdu University, Chengdu 610106, China; d College of Food Science and Nutritional Engineering, China Agricultural University, Beijing 100083, China

**Keywords:** *Phyllanthus emblica L*, Yeast strains, Chemometrics, Fermentation

## Abstract

Since the key aroma compounds and bioactive profiles of *Phyllanthus emblica* fruit wine (PFW) driven by different yeast strains remain unclear, this study aimed to systematically evaluate the effects of five *Saccharomyces cerevisiae* strains on physicochemical properties, bioactive components, and volatile organic compound (VOC) profiles in PFW. Using an integrated flavoromics approach with chemometric analysis, we identified 110 VOCs. FR and D254 enhanced ester concentrations and antioxidant activity respectively, whereas strain L2323 significantly altered aroma complexity by promoting specific esters and alcohols. Multivariate analyses identified methyl nitrate and n-propyl acetate as discriminant markers among strains. Aroma-activity analyses further highlighted ethyl hexanoate, isoamyl acetate, and n-propyl acetate as major aroma contributors with distinctive strain-dependent distributions. These findings provide critical guidance for yeast strain selection and fermentation optimization to improve PFW quality, offering new insights for the development of functional fruit wines.

## Highlights

• Integrated HS-SPME-GC–MS and HS-GC-IMS enabled comprehensive characterization.
• Distinct yeast strains modulated the aroma and bioactive profiles of PFW.
• Ester-rich fermentations correlated with higher antioxidant activity and quality.
• Key aroma-active compounds, including ethyl acetate were strain-dependent.

## 1 Introduction

*Phyllanthus emblica L*., belonging to the *Euphorbiaceae* family and commonly known as Indian gooseberry or yuganzi in China, is widely recognized for its exceptional nutritional and medicinal value, owing to its rich content of polyphenols, flavonoids, vitamin C, and tannins (Gul et al., 2022; Li, Liu et al., 2020; Wang et al., 2019). It is well established that oxidative stress caused by free radicals can damage biological macromolecules such as DNA, lipids, and proteins, ultimately leading to cellular dysfunction (Ito et al., 1986). These oxidative interactions are closely linked to the progression of chronic conditions, including cardiovascular diseases, cancer, and aging-related disorders. *P. emblica* fruit (PF) is rich in bioactive compounds with strong antioxidant properties, which can effectively scavenge free radicals and inhibit oxidative chain reactions, thereby offering protective effects against oxidative damage (Li et al., 2008). Traditionally used in Eastern medicine and functional foods, PF has recently garnered growing interest as a raw material for the development of value-added fermented products, with the demand for functional foods among consumers (Vivek et al., 2019). However, its naturally sour, astringent, and pungent taste presents challenges in terms of flavor acceptability, limiting the application in the food and beverage industry. These sensory drawbacks have become a major obstacle to the large-scale industrialization of PF-based products. Exploring fermentation strategies, particularly fruit wine production, offers a promising approach to not only enhance the nutritional value of PF but also improve its sensory characteristics and overall consumer acceptance.

Fermented fruit wines are products derived from the complex interactions between microorganisms and natural fruits rich in carbohydrates. Recent studies have highlighted the pivotal role of strains such as yeasts (Velenosi et al., 2021), *Acetobacter* (Paz-Arteaga et al., 2023), and *Lactobacillus* (Meng et al., 2022) in the fermentation of fruits. In addition, these microorganisms promote the production of bioactive compounds through diverse metabolic pathways, thereby enhancing the antioxidant and antimicrobial properties of the final products. On the other hand, the microbial communities generated during fermentation could modulate the gut microbiota and contribute to the formation of beneficial metabolites, such as short-chain fatty acids (SCFAs) and γ-aminobutyric acid (GABA) (Ma et al., 2021). The aroma profile and sensory characteristics of fermented fruit wine are also highly dependent on yeast strains selection (Lin et al., 2019). Different yeast strains exhibit distinct metabolic activities, leading to the production of varying metabolites that ultimately influence the flavor attributes of the final product (Sherman et al., 2020). Lin et al. (2019) investigated the fermentation of pineapple and *Syzygium samarangense* wines using four commercial yeast strains and found significant differences in their capacities to synthesize and release volatile compounds. Strains D254 and BV818 were more suitable for pineapple wine, whereas D254 and RV100 generated the highest diversity of volatiles in *S. samarangense* fermentation. Additionally, studies have shown that *Zygosaccharomyces rouxii* and *Saccharomyces cerevisiae* display specific abilities in modulating polyphenol content and the formation of various aroma-active compounds when applied to the fermentation of different fruit wines (Kokkinomagoulos et al., 2020). Therefore, exploring the role of *S. cerevisiae* in the development of flavor and bioactive compounds is essential for promoting the development of *P. emblica*-based functional products.

Since the wine yeast concept of flavoromics was proposed in 2008, it has marked a significant advancement in the characterization of food flavor components. These chemical constituents can be effectively integrated with chemometric methods or deep learning algorithms to screen and identify key flavor compounds (Cai et al., 2024). Chromatography–mass spectrometry techniques (GC–MS and GC-IMS) and intelligent sensory technologies (electronic nose and electronic tongue) are commonly used analytical tools in flavoromics (Yuan et al., 2025). The former offers advantages such as high sensitivity and rapid analysis, while the latter simulate human olfactory and gustatory perception and provide more objective and accurate evaluations compared to traditional sensory analysis. Therefore, integrating chromatographic-mass spectrometry techniques with intelligent sensory analysis bridges objective instrumental data with subjective sensory evaluation, enabling a more comprehensive characterization and understanding of the relationship between food flavor and chemical composition. This approach facilitates the identification of key flavor attributes and the elucidation of flavor formation mechanisms.

This study aims to provide preliminary insights into the application of PF in functional fermented foods. Five commercial strains of *S. cerevisiae* (BV818, FR, SY, L2323, and D254) were selected as fermentation starters for *P. emblica* fruit wine (PFW) production. Organic acids were quantified using liquid chromatography, while volatile flavor compounds were systematically characterized through a flavoromics approach involving headspace gas chromatography-ion mobility spectrometry (HS-GC-IMS), headspace solid-phase microextraction gas chromatography–mass spectrometry (HS-SPME-GC–MS), electronic nose, and electronic tongue analyses. The contribution of key aroma-active compounds was further evaluated using relative odor activity value (ROAV) and odor activity value (OAV) calculations.

## 2 Materials and methods

### 2.1 Materials

Fresh PF were purchased from Jieyang, Guangdong Province, China. All five yeast strains (BV818, FR, SY, L2323, and D254) were single-strain commercial active dry yeasts of *Saccharomyces cerevisiae* purchased from Jinan Shuangmai Beer Materials Co., Ltd. According to supplier technical sheets and published datasheets, the panel spans distinct phenotypes—robust/high-tolerance (BV818, SY), structure-oriented with enhanced phenolic extraction (L2323), and balanced aroma–mouthfeel (D254). Public documentation for FR was limited, therefore, no a priori phenotype was assumed for FR, and its fermentation behavior was treated as unknown prior to this work and evaluated empirically in the present study under identical conditions. Starters were stored at 4 °C.

Sodium carbonate (Na_2_CO_3_), phenolphthalein, sodium hydroxide (NaOH), 1,1-diphenyl-2-picrylhydrazyl (DPPH), anhydrous ethanol, salicylic acid, ferrous sulfate (FeSO_4_), hydrogen peroxide (H_2_O_2_), rutin, potassium bicarbonate (KHCO_3_), aluminum nitrate [Al(NO_3_)_3_], gallic acid, Folin–Ciocalteu reagent, and methanol were all analytical grade (≥99 % purity) and were purchased from Shanghai Aladdin Biochemical Technology Co., Ltd. (Shanghai, China). Organic acid standards—malic acid, acetic acid, citric acid, succinic acid, and fumaric acid were provided by MO Quality Inspection Technology Co., Ltd. (Changzhou, China). Ultrapure water was used throughout the experiments.

### 2.2 Preparation of PFW

Upon arrival at the laboratory, all fresh PF selected for the study were quickly washed, peeled, and pressed to obtain the juice. To promote the release of volatile organic compounds (VOCs) and standardize sugar content for subsequent fermentation, the juice was diluted 1:1 with deionized water following Zhou et al. (2023). Each fermentation had a working volume of 200 mL. Sucrose was added to achieve a final sugar concentration of 23°Brix. To prevent browning, potassium metabisulfite (70 mg/L) and citric acid were added. Pectinase (0.02 g/L, 100,000 U/g; SAS SOFRALAB, France) was subsequently added to promote the release of cellular contents. The mixture was incubated at 40 °C for 2 h in a thermostatic water bath, after which the pH was adjusted to 4.0 using citric acid or calcium carbonate. Five yeast strains (0.2 g/L each) were inoculated separately, and fermentation was carried out at 25 ± 1 °C for 14 days until soluble solids (°Brix) stabilized. Each yeast strain was fermented in triplicate. Upon completion of fermentation, PFW was filtered through sterile triple-layer gauze and clarified by refrigeration at 4 °C for 1 week. The supernatant was subsequently collected for analytical assays.

### 2.3 Physicochemical parameters in PFW

Alcohol content and titratable acidity were determined according to GB/T 15038–2006. The total phenolic content (TPC) was assessed following de Lima et al. (2024), with minor adjustments. Using the Folin–Ciocalteu reagent and using gallic acid as a reference standard, the total phenolic content was quantified in various treatment groups via spectrophotometry. Specifically, 1 mL of PFW was combined with 9 mL of distilled water in a 25 mL volumetric flask. Afterward, 1 mL of Folin–Ciocalteu reagent was added and the mixture was allowed to react for 6 min. Following this, 3 mL of 7 % sodium carbonate solution was added and the mixture was brought up to the 25 mL mark with distilled water to complete the volumetric preparation. A reagent blank was also prepared using distilled water. The mixture was left to incubate in the dark for 60 min to facilitate the reaction and then the absorbance was measured at 760 nm using a multiplate reader (Synergy, BioTek, Vermont, USA). A calibration curve was established with gallic acid as the reference standard to determine the total phenolic content (mg/L).

For the quantification of total flavonoids, the procedure was modified based on the method described by de Lima et al. (2024). Briefly, 1 mL of the PFW was pipetted into a 10 mL volumetric flask, followed by the addition of 0.3 mL of a 5 % sodium nitrite solution and 4 mL of distilled water. This mixture was then incubated for 5 min before the introduction of 0.3 mL of a 10 % aluminum chloride solution, and the mixture was allowed to react for 6 min. Then 2 mL of 1 mol/L NaOH was added, and the volume was made up to 10 mL with distilled water. After incubating for 30 min, the absorbance at 510 nm was measured using a multiplate reader (Synergy, BioTek, Vermont, USA). A standard curve using rutin as the reference compound was constructed to calculate the flavonoid content (mg/L).

For DPPH radical-scavenging activity (de Lima et al., 2024), 0.005 g of DPPH was dissolved in anhydrous ethanol and diluted to 250 mL to prepare the DPPH working solution. Experimental protocol for DPPH assay: (a) Prepare a blank by mixing 2 mL of DPPH anhydrous ethanol solution with 2 mL of anhydrous ethanol, labeled V0. (b) For the initial sample absorbance, mix 2 mL of DPPH anhydrous ethanol solution with 2 mL of the sample solution, labeled Vi. (c) For the sample blank, mix 2 mL of anhydrous ethanol with 2 mL of the sample solution, labeled Vj. All mixtures were incubate in the dark at room temperature for 30 min, and absorbance was measured at 517 nm. Assay were conducted in triplicate to ensure reliable results. The calculation formula was:

(0.1) $$\mathrm{DPPH}\;\mathrm{clearance}\begin{array}{cc} & \mathrm{rate}=1-\frac{\mathrm{Vi}-\mathrm{Vj}}{\mathrm{Vo}} \end{array}*100\%$$

*The ABTS radical scavenging activity was measured with slight modifications based on the method of* *Chen et al. (*2023*)**. Briefly, 1 mL of sample was mixed thoroughly with 4 mL of ABTS solution (7.4 mM/L). The mixture was incubated in the dark at room temperature for 10 min. The absorbance was measured at 734 nm. The ABTS scavenging rate was calculated as:*

(0.2) $$\mathrm{ABTS}\left(\%\right)=\frac{\mathrm{AO}-A}{\mathrm{AO}}*100\%$$

where A is the absorbance of the sample and A₀ is the absorbance of the control, in which 95 % ethanol was used instead of the sample.

### 2.4 Analysis of the organic acids

Organic acids in PFW were analyzed using high-performance liquid chromatography (HPLC). Prior to analysis, the supernatant was filtered through a 0.22 μm membrane filter (Shandong Bonana Biotechnology Co., Ltd., Shandong, China). Chromatographic separation was performed on an Agilent C18 column (4.6 mm × 250 mm, 5 μm). The mobile phase consisted of solvent A (0.1 % phosphoric acid in water) and solvent B (methanol). The gradient program was: 0–2.0 min, 97.5 % A (2.5 % B); 2.0–4.0 min, linear to 0 % A (100 % B); 4.0–6.0 min, 0 % A; 6.0–8.0 min, linear back to 97.5 % A; 8.0–10.0 min, 97.5 % A for re-equilibration. The flow rate was set at 0.5 mL min^−1^, with the column temperature maintained at 35 °C. The injection volume was 10 μL, and the total run time was 10 min. Detection was carried out at 210 nm. Organic acids were identified and quantified against external standards by retention time and peak area (calibration curves), and results were expressed in mg/L.

### 2.5 Electronic sensory assay

*Taste analysis was performed using an α-ASTREE electronic tongue system (Alpha MOS, Toulouse, France), equipped with a five-sensor array and two reference electrodes to detect key taste attributes: sourness (AHS), saltiness (CTS), umami (NMS), bitterness (SCS), and sweetness (ANS). Prior to measurement, 5 mL of PFW was diluted to 80 mL (deionized water). The instrument operated under controlled conditions with a data acquisition time of 120 s, an acquisition cycle of 1.0 s, and a stirring speed of 1 rev s* ^*−1*^ *.*

*A FOX 4000 electronic nose system (Alpha MOS, Toulouse, France), equipped with 18 non-specific metal oxide sensors, was used for the analysis of volatile organic compounds (VOCs) in PFW. Each sensor type exhibits distinct sensitivity to different classes of volatiles. For analysis, 5 mL of sample was placed in a 10 mL headspace vial and incubated at 70 °C for 300 s to facilitate headspace equilibration. Then, 500 μL of headspace gas was extracted at a rate of 1500 μL s* ^*−1*^ *. Data acquisition was conducted at a flow rate of 150 mL min* ^*−1*^ *, with a cycle time of 1.0 s and a total acquisition time of 120 s.*

### 2.6 Analysis of VOCs in PFW by HS-GC-IMS

Volatile compounds were analyzed using HS–GC–IMS system equipped with an autosampler and a heated gas-tight syringe. A WAX capillary column (30 m × 0.53 mm i.d., 1.0 μm film thickness; Restek, Bellefonte, PA, USA) was used for chromatographic separation. For analysis, 5 mL of sample was transferred into a 20 mL headspace vial and incubated at 65 °C for 15 min. Subsequently, 400 μL of headspace gas was injected at 85 °C in splitless mode. Chromatographic separation was carried out under isothermal conditions at 60 °C using nitrogen (≥99.999 % purity) as the carrier gas. The carrier-gas flow was programmed as follows: 2 mL min^−1^ for 2 min; ramp to to 10 mL min^−1^ over 8 min; ramp to 100 mL min^−1^ over 10 min; ramp to 150 mL min^−1^ over 10 min. The IMS drift tube was operated at 45 °C. Volatile compounds were tentatively identified by matching retention index (RI) and drift time (DT) against the built-in HS–GC–IMS library within LAV/VOCal (with RI cross-referenced to NIST where applicable).

### 2.7 Analysis of VOCs in PFW by HS-SPME-GC–MS

Volatile compounds were also analyzed using an SQ680 GC–MS (PerkinElmer, Inc., Waltham, MA, USA). Briefly, 5 mL of PFW was transferred into a headspace vial, and 20 μL of 2-methyl-3-heptanone (5 μg/mL) was added as an internal standard. Headspace extraction was performed using solid-phase microextraction (SPME) fiber at 45 °C for 10 min, followed by desorption at 250 °C for 5 min in the GC–MS inlet, with an Elite-5MS column installed (30 m × 0.25 mm × 0.25 μm; Perkin Elmer Technologies Inc.). Helium was used as the carrier gas at a flow rate of 1.0 mL min^−1^, with splitless injection. The oven temperature was initially held at 40 °C for 2 min, then to 80 °C at 3 °C min^−1^ (5 min), to 105 °C at 2 °C min^−1^ (5 min), to 180 °C at 3 °C min^−1^, and to 230 °C at 10 °C min^−1^ (5 min). Mass spectra were acquired in EI mode (70 eV; scan range *m*/*z* 40–600), with quadrupole and ion source temperatures maintained at 150 °C and 230 °C, respectively (Li, Huang, et al., 2024). Volatile compounds were quantified based on peak areas relative to the internal standard. Compound identification was based on comparison of acquired mass spectra with the NIST 2014 library (and PubChem for supplementary reference, where applicable). Each compound was accepted at a library match score ≥ 800 together with the corresponding retention time (RT).

### 2.8 Calculation of OAV and ROAV

OAV, as described by Guo et al. (2022), is the ratio of the semi-quantitative concentration of a compound to its odor threshold.

(3) $${\mathrm{OAV}}_{i}=\frac{C_{i}}{{\mathrm{OT}}_{i}}$$

where C_i_ and OT_i_ represent the concentration and odor threshold of compound i, respectively.

The formula for ROAV, as described by Yuan et al. (2025), is as follows:

(4) $${\mathrm{ROAV}}_{i}=\frac{C_{i}}{T_{i}}\times \frac{T_{\max}}{C_{\max}}\times 100$$

where T_i_ and C_i_ represent the threshold and concentration values, respectively, and T_max_/C_max_ denotes the maximum ratio of C_i_/T_i_ among all compounds in the SV sample.

### 2.9 Statistical analysis

The statistical analysis was performed using R (version 4.4.3). All data were, as needed, transformed to meet normality assumptions prior to univariate analyses. ANOVA followed by Tukey's HSD post hoc test (*p* < 0.05) was used to determine significant differences. Visualizations were prepared using Origin 2021 (OriginLab, USA) and Chiplot Online (https://www.chiplot.online/). Multivariate statistical methods, including principal component analysis (PCA) and orthogonal partial least squares discriminant analysis (OPLS-DA), were performed in SIMCA 14.1 (Umetrics, Sweden). Network relationships were visualized in Cytoscape (version 3.0) using a force-directed layout.

## 3 Results and discussion

### 3.1 Physicochemical properties and antioxidant activity of PFW

The PFW samples fermented with each of the five yeast strains reached dryness, but significant strain-dependent differences were observed in ethanol yield and acidity (ANOVA: ethanol F(4,10) = 144.57, *p* < 0.001; acidity F(4,10) = 9.73, *p* = 0.0018). Fermentation with strain D254 produced the highest alcohol content (%*v*/v), whereas FR yielded the lowest (D254 > SY/FR/L2323/BV818; all *p* ≤ 0.001; FR ≈ SY, *p* = 0.806). All fermentations proceeded to completion under the same conditions, indicating similar sugar-consumption kinetics; thus, compositional differences arise from yeast metabolic activity rather than incomplete fermentation. These findings are consistent with known strain-specific fermentation efficiencies; different *S. cerevisiae* strains have distinct sugar-assimilation and ethanol-production rates (Loira et al., 2013). Some strains divert more carbon to byproducts (glycerol or organic acids) at the expense of ethanol, which can explain variations in final alcohol content. Indeed, yeasts often trade off glycerol synthesis against ethanol yield. Glycerol production drains sugar carbon; therefore, a more glycerogenic strain would deliver lower alcohol. Similarly, titratable acidity differed among wines: D254 (4.75 ± 0.19 g/L) > SY (4.05 ± 0.15; *p* = 0.00117), and FR/L2323 > SY (*p* = 0.00869/0.0127); other contrasts were not significant. Acidity is a key factor influencing the taste of fruit wines; moderate acidity helps preserve fruity aromas and balance sweetness and sourness, thereby enhancing overall palatability (Zhang et al., 2017). Such acidity variations likely reflect strain-dependent production of organic acids during fermentation.

### 3.2 Effect of enzymolysis on antioxidant capacity of PFW

**Fig. 1:**
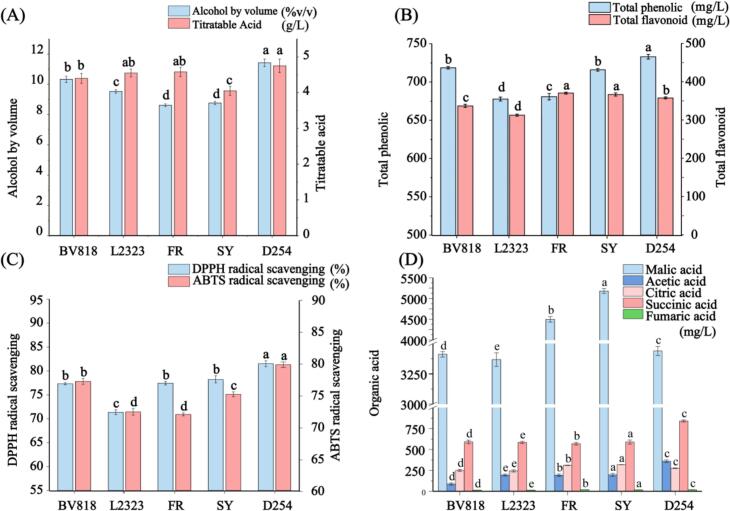
Physicochemical indicators and organic acids of PFW fermented by different yeasts. Note: (A) Alcohol by volume (%*v*/v) and titratable acidity (TA, g/L as tartaric acid equivalents), (B) The total phenolic concentration (mg/L) and total flavonoid concentration (mg/L), (C) antioxidant activities based on DPPH (%) and ABTS (%) of PFW samples, (D) content of five organic acids (mg/L) fermented with different yeast strains. Values represent the mean ± standard deviation of triplicate experiments.

More striking were the differences in polyphenolic bioactives and antioxidant capacity. As shown in Fig. 1B, TPC differed by strain (F(4,10) = 237.42, *p* < 0.001) with D254 highest; TFC likewise differed (F(4,10) = 156.97, *p* < 0.001) with FR/SY highest and > D254 (FR *p* = 0.0045; SY *p* = 0.0439).These strain-dependent differences in polyphenol levels likely reflect variation in yeast metabolism and enzyme secretion during fermentation. Beyond generic enzyme effects, the interaction between the polyphenol matrix of PF and yeast metabolism is decisive: PF is dominated by hydrolysable tannins (ellagitannins, gallotannins), together with gallic/ellagic acids and flavonol O-glycosides (quercetin- and kaempferol-derived glycosides) (Prananda et al., 2023). During *S. cerevisiae* fermentation, tannase and esterase activities can hydrolyze galloyl/HHDP (hexahydroxydiphenoyl) esters, increasing free gallic/ellagic acids (Dos Anjos et al., 2024); β-glucosidase can cleave flavonol O-glycosides, releasing aglycones that typically exhibit higher antioxidant capacity but stronger bitterness/astringency (Zhang et al., 2021). In parallel, yeast cell-wall mannoproteins/β-glucans may adsorb high-molecular-weight tannins, thereby decreasing measurable TPC/TFC in a strain-dependent manner (Nguela et al., 2023). The acidic pH and rising ethanol further promote hydrolysis/solubilization, while the ascorbate-rich PF matrix can help maintain phenolics in reduced forms (Prananda et al., 2023).

While higher TPC/TFC strengthens antioxidant capacity (Gebicki & Nauser, 2021), it may also intensify perceived astringency and, to a lesser extent, bitterness. Mechanistically, condensed tannins and selected flavonoids can bind proline-rich salivary proteins, promote protein–polyphenol aggregation/precipitation, and reduce oral lubrication, thereby increasing dryness/roughness. Within the present dataset, the D254 fermentation—showing the highest TPC—therefore carries a higher astringency risk than FR or L2323, despite its antioxidant advantage. Practically, this trade-off can be managed through post-fermentation strategies (e.g., short maturation to soften tannins, protein or PVPP fining where permitted, mannoprotein addition, or blending) implemented only if astringency exceeds target. Because no trained panel data were collected here, these are mechanistic predictions.

Consistent with this, we assessed the antioxidant activity of each PFW sample using DPPH and ABTS radical-scavenging assays. Fig. 1C illustrates the antioxidant activities of PFW samples fermented with five different yeast strains. The wine fermented with strain D254 showed the highest DPPH scavenging activity (81.50 %, *p* < 0.05), significantly above that of all other strains. In contrast, the L2323-fermented wine exhibited the lowest DPPH activity, while the BV818, FR and SY did not differ (*p* = 0.753, 0.062, 0.106). A similar pattern was observed in the ABTS assay (F(4,10) = 187.39, *p* < 0.001): D254 yielded the highest scavenging (79.72 %), whereas L2323 (72.35 %) and FR (71.94 %) were lowest, and BV818 (77.11 %) and SY (75.08 %) were intermediate. This matches established findings that higher phenolic content yields greater antioxidant capacity (Li et al., 2017). Altogether, these results indicate that D254's strain-specific enzymatic profile confers a significant advantage, yielding PFW with superior TPC, TFC and antioxidant capacity.

### 3.3 Analysis of the organic acids in PFW

The content of organic acids is crucial in assessing the flavor, and overall quality of fruit-fermented beverages (Fejzullahu et al., 2021). In this study, the organic acid profiles of PFW samples fermented with different yeast strains were analyzed, and five major organic acids were identified (Fig. 1D): malic acid, acetic acid, citric acid, succinic acid, and fumaric acid. Among them, malic acid was the predominant component, with concentrations significantly higher than the others. Specifically, the malic acid levels in the five samples were ranked as follows: SY (5179.03 mg/L) > FR (4497.48 mg/L) > D254 (3439.64 mg/L) > BV818 (3412.51 mg/L) > L2323 (3368.33 mg/L). Previous research has shown that malic acid plays a central role in yeast metabolism as a key intermediate in the glyoxylate and tricarboxylic acid (TCA) cycles (Qin et al., 2023). The high malic acid content observed may be attributed to the naturally high levels of malic acid in PF (1.4–3.8 % by weight) (Tewari et al., 2023) combined with the limited malic acid degradation capacity of *S. cerevisiae*, which inherently lacks a dedicated malate transporter and has low-affinity mitochondrial malic enzymes (Volschenk et al., 1997). Notably, lactic acid was not detected in any of the samples (detection limit <4.09 mg/L). Since LAB proliferation generally occurs in the late stages of alcoholic fermentation, the 14-day fermentation period combined with the naturally low pH of *P. emblica* likely created an unfavorable environment for LAB growth. In addition, the use of pure *S. cerevisiae* inocula without bacterial contamination minimized the possibility of lactic acid production, as *S. cerevisiae* strains inherently lack the ability to synthesize lactic acid. Aside from malic acid, the concentrations of other organic acids were relatively low. In the D254 group, acetic acid was the third most abundant organic acid; when present at concentrations near its sensory threshold (0.7–1.1 g/L), it can impart a mild vinegar-like aroma to the wine (Lambrechts & Pretorius, 2000). In contrast, in the other yeast-fermented samples, citric acid was the third predominant acid, with no significant differences among samples. Additionally, fumaric acid, which contributes sourness, astringency, and umami, showed comparable concentrations between BV818 and L2323 and between FR and D254.

### 3.4 Analysis of the differences in flavor profiles of PFW from different yeasts by intelligent sensory instrument evaluation

#### 3.4.1 *E*-nose analysis

**Fig. 2:**
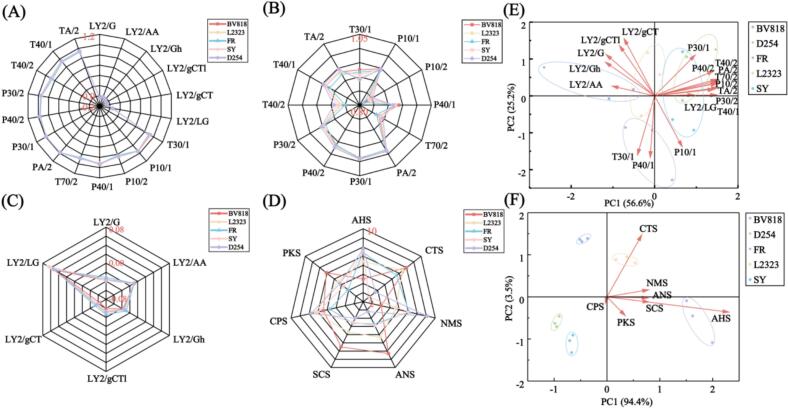
Variation of response characteristics of PFW from five different yeast strains by E-nose and E-tongue. Note: (A) Radar plot of all sensor response values for E-nose, (B) Radar plot of 12 primary sensor response values for E-nose, (C) Radar plot of 6 secondary sensor response values for E-nose, (D) PCA of VOCs biplot for E-nose, (E) Radar plot of all sensor response values for E-tongue, (F) PCA of non-volatile taste biplot for E-tongue.

Sensors on the e-nose exhibited patterns that map directly onto chemical classes of PFW. As shown in Fig. 2A, PFW of different yeast strains triggered 18 sensors to respond, but the response strength varied. Among them, Fig. 2B indicates that sensors PA/2, P30/1 and T40/1 showed the largest signals; these sensors are broadly sensitive to organic volatiles (Table S1), and thus would respond strongly to fermentation alcohols (ethanol) and sulfur-containing volatiles such as furfuryl mercaptan (a thiol). These compounds might have an essential influence on the PFW aroma structure (Li, Zhang, et al., 2024). Indeed, metal-oxide sensors can exhibit dose-dependent responses to ethanol (Yuan et al., 2024), so elevated readings on PA/2 and P30/1 likely reflect higher alcohol content in some FPW samples, which was broadly consistent with the alcohol trend above. Other sensors target esters and aromatics: for instance, LY2/gCT and T70/2 are tuned to aromatic hydrocarbons; P10/2 to aliphatic compounds. Consistently, “fatty acid” esters, which give fruity and floral notes, are known to dominate e-nose responses in fermented beverages (Li et al., 2023), so variation in ester production by yeast would contribute to the observed patterns. Principal-component analysis (PCA) of the 18-sensor array thus segregated the five PFW samples (Fig. 2E-F) into distinct clusters. Each cluster's position in PCA space can be interpreted by its sensor loadings. A strain that produces relatively more higher alcohols would score toward the PA/2 and P30/1 axes (the “alcoholic” direction), whereas a strain richer in amine or aromatic volatiles would shift toward the LY2/gCTI or LY2/gCT axes. This clustering indicated that PFW fermented with different yeast strains had distinct aroma structures. (Li, Guo, et al., 2024). Accordingly, we believe that each yeast strain imparts a characteristic balance of alcohols, esters, and aromatics in the PFW, and these chemical differences underlie the aroma structure distinctions captured by the PCA.

#### 3.4.2 E-tongue analysis

The E-tongue, utilizing artificial lipid membrane technology, enables the digital evaluation of aftertaste by detecting key taste characteristics such as sourness, sweetness, bitterness, umami, and saltiness (Wei et al., 2020). As shown in Fig. 2D, the sample fermented with D254 exhibited the highest levels of sweetness (7.8) and umami (9.1). In contrast, BV818 showed the strongest intensities in saltiness (8.0), sourness (8.3), and bitterness (7.5); these three taste attributes are generally regarded as negatively correlated with wine quality (Cosme et al., 2021). Furthermore, FR and SY displayed similar taste profiles, with all taste intensities falling between those of D254 and BV818. Previous studies have shown that phenolic and heterocyclic molecules predominantly contribute to bitterness and astringency, while reducing amino acids, sugars, and their derivatives affect perceptions of freshness and saltiness (Gong et al., 2021; Zhang et al., 2019). Presumably, variations in these volatile and non-volatile compounds are among the main factors driving the observed taste differences in PFW. Subsequently, a classification model linking taste attributes and yeast strains was constructed using PCA (Fig. 2F). The first two principal components, PC1 (94.4 %) and PC2 (3.5 %), together explained 97.9 % of the total variance, indicating that they effectively captured the essential differences in the dataset. Based on this, it can be concluded that the taste characteristics of PFW varied significantly depending on the yeast strain, consistent with the e-nose findings on chemical classes differentiation. This also emphasized the importance of further investigating the interaction between aroma and taste to gain a more comprehensive understanding of PFW's overall flavor profile.

### 3.5 Characterization of VOCs by HS-GC-IMS

**Fig. 3:**
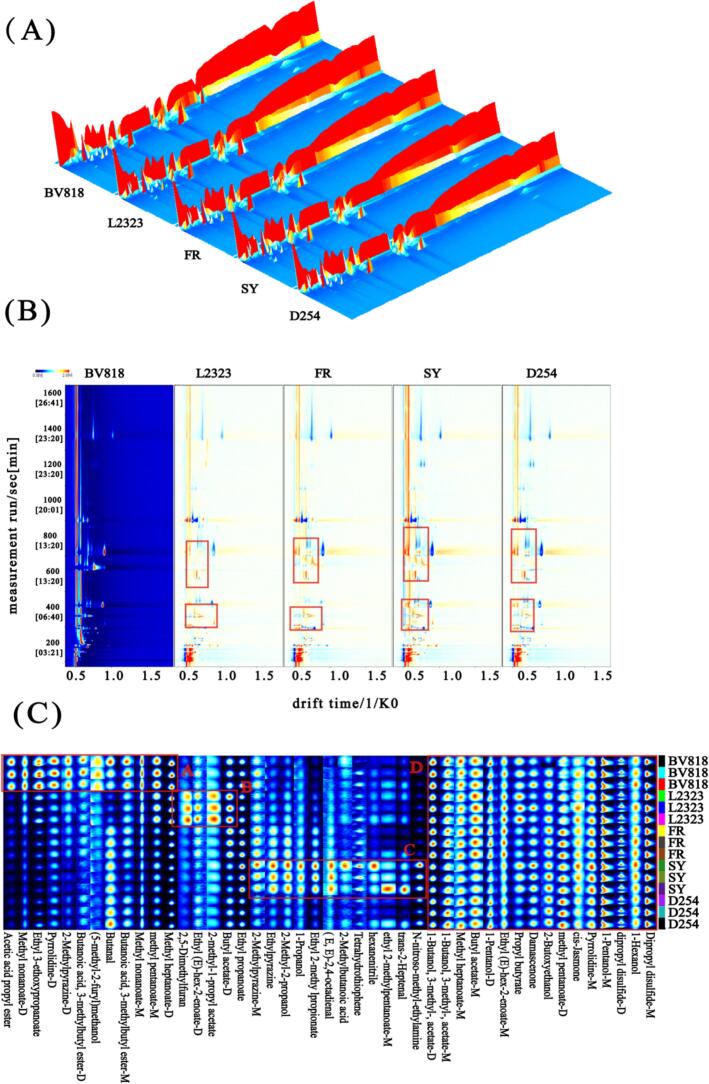
Spectra of volatile components of PFW samples obtained by HS-GC-IMS method. Note: (A) 3D topographic map, (B) 2D spectrogram of difference comparison spectrogram (BV818 as reference). The X-, Y-, and *Z*-axes represent retention time, ion drift time, and signal intensity, respectively. Red indicates a higher concentration of the compound relative to the reference sample, while blue indicates a lower concentration, (C) VOCs fingerprint chromatogram. Each row represents an PFW sample and each column a volatile compound. The color scale from blue to red indicates relative intensity, with red denoting higher abundance. (For interpretation of the references to color in this figure legend, the reader is referred to the web version of this article.)

The dynamic changes in volatile abundance reflect the evolution of PFW's flavor profile at the molecular level (Andreou et al., 2023). In this study, HS-GC-IMS was used to analyze the VOC composition of PFW fermented with different yeast strains, focusing on how yeast metabolism influences aroma. A total of 45 VOCs were identified in the PFW (Table S2). It should be noted that some substances may be detected as monomers and dimers due to high substance concentrations or strong intermolecular forces during the ionization process (Li et al., 2025). These compounds comprised 21 esters, 12 heterocyclic compounds, 2 ketones, 6 alcohols, 3 aldehydes, and 1 acid, representing the major aroma-contributing categories in the fermentations. Esters dominated the profile, followed by notable contributions from heterocycles and aldehydes. We first visualized the VOC profile evolution using a 3D topographic plot of drift time, retention time, and signal intensity (Fig. 3A). The 3D spectra revealed clear differences between yeast strains: PFW samples fermented with other yeast strains showed a greater number and higher intensity of ion signal peaks than the D254-fermented sample, suggesting more abundant volatile compounds in those fermentations. To facilitate direct comparison of VOC distributions, we then projected the data into a two-dimensional fingerprint view (Fig. 3B) with the BV818 fermentation as a reference. In this top-view plot, the red line denotes the reactant ion peak (RIP), serving as an internal standard for compound identification (Zhang et al., 2024). Notably, fermentations with other yeasts exhibited a higher density of red signal dots in the drift time range 0.5–1.0 ms and retention time 400–800 s compared to BV818. This indicates the presence of more volatile compounds at higher concentrations in that region of the spectrum for those strains. In addition, several blue signal spots appeared in the 800–1000 s retention time region for the non-BV818 samples, suggesting the existence of certain volatiles at lower concentrations or with weaker/negative ion mobility responses in those wines. These differences in the distribution and intensity of volatile signals reflect the distinct metabolic activities of each yeast strain, which modulate the formation or degradation of key aroma metabolites during fermentation (Guan et al., 2025; Zhang, Ma, et al., 2023). The HS-GC-IMS flavor fingerprint (Fig. 3C) and Table S2 confirmed strain-specific VOC differences, allowing us to identify distinctive VOCs for each yeast treatment (*p* < 0.05). PFW fermented with strain BV818 was characterized by ester compounds such as acetic acid propyl ester, methyl nonanoate-D, and ethyl 3-ethoxypropanoate, as well as nitrogen-containing heterocycles including pyrrolidine-D and 2-methylpyrazine-D. These compounds were significantly higher in the BV818 sample, indicating this yeast's metabolism favors the production of certain esters and heterocyclic aroma molecules. In the L2323 fermentation, the distinctive VOCs were 2,5-dimethylfuran (a heterocyclic furan) and the esters (*E*)-ethyl 2-hexenoate-D and 2-methyl-1-propyl acetate. Meanwhile, strain SY produced different volatiles: ethyl 2-methylpropionate, (E,E)-2,4-octadienal and trans-2-heptenal, and a nitrile (hexanenitrile). The presence of these aldehydes and the nitrile in SY's profile implies that this yeast's metabolism may lead to greater accumulation of certain oxidative or amino acid breakdown products, imparting sharper green or fatty aroma nuances compared to the other strains.

### 3.6 Characterization of VOCs in PFW by HS-SPME-GC–MS

**Fig. 4:**
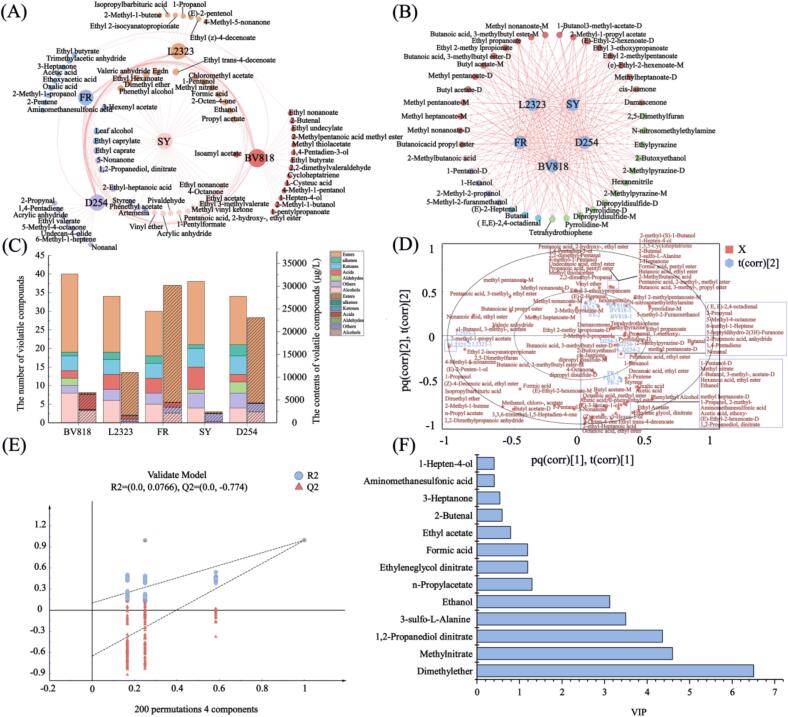
Multivariate analysis and total VOCs analysis in PFW with five yeast strains. Note: (A) Flavor distribution profile of HS-SPEM-GC–MS, (B) Flavor distribution profile of HS-GC-IMS, (C) Volatile compound contents (oblique line) and distribution across different types (solid color) in PFW, (D) OPLS-DA biplot showing clustering of PFW samples based on VOC profiles, (E) result of 200 permutations tests, (F) VIP score chart.

To comprehensively profile the flavor characteristics of PFW, HS-SPME-GC–MS was conducted as a quantitative complement to HS-GC-IMS analysis (Wei et al., 2023; Yuan et al., 2025). Using HS-SPME-GC–MS, 71 volatile compounds were identified (Fig. 4), comprising esters (26), alcohols (11), aldehydes (6), ketones (7), acids (9), alkenes (6), and others (6). Consistent with HS-GC-IMS results, esters were the dominant volatile class. In total, 110 unique VOCs were identified across all samples by integrating HS-SPME-GC–MS and HS-GC-IMS analyses (Table S3). Among these, four compounds (1-propanol, 1-pentanol, propyl acetate, and isoamyl acetate) were detected by both techniques, whereas 67 VOCs were exclusively identified via HS-SPME-GC–MS and 41 exclusively via HS-GC-IMS. HS-SPME-GC–MS exhibited superior sensitivity in detecting esters and acids, while HS-GC-IMS uniquely identified heterocyclic VOCs such as pyrrolidine, 2-methylpyrazine, and ethylpyrazine. This complementary analytical approach aligns well with recent findings on fruit wine VOC profiling (Xi, Zhang et al., 2024), which will be more beneficial for exploring the influence of different yeasts on PFW flavor.

The general situation of volatile flavor substances across different samples was analyzed in greater detail using a force-directed layout diagram. As shown in Fig. 4A and B, the diversity and abundance of VOCs varied among yeast strains, highlighting unique aroma profiles generated by each strain (He et al., 2023). Fig. 4C presents a stacked histogram illustrating the evolution of quantity and content of volatile flavor substances in different PFW. A clear strain-dependent variation was observed in ester profiles. In comparison to the others, the FR-fermented wine exhibited the highest total ester concentration (23,984 μg/L), followed by D254 (18,695 μg/L) and L2323 (9361 μg/L), resulting in enhanced sensory characteristics characterized by a desirable and fruity full-bodied ester aroma (Muñoz-Redondo et al., 2021). Specifically, among the major esters identified were ethyl acetate, ethyl isovalerate, and isoamyl acetate, with ethyl acetate being both the most characteristic and the most abundant aroma compound (Zhang, Qin, et al., 2023). Ethyl hexanoate, a compound associated with apple- and apricot-like aromas, was present at a concentration of 8.91 μg/L in the FR-fermented sample, significantly exceeding the levels detected in BV818 and L2323 samples (2.7–2.8 μg/L), and was barely detectable in the SY sample (0.30 μg/L). Indeed, yeast strains differ in their ester-forming capabilities, potentially linked to specific enzyme activities like ethanol hexanoyl transferase (Eht1p/Eeb1p) involved in ethyl hexanoate synthesis (Lilly et al., 2006; Yang et al., 2022a, Yang et al., 2022b). It is speculated that strain-dependent variation in the expression or activity of these genes contributes to the observed differences in ester production. For example, a comparative fermentation of an indigenous wine yeast (strain Z622) versus a commercial strain found that Z622 produced significantly more acetate esters and concomitantly had much higher ATF1/ATF2 transcript levels (Parapouli et al., 2019). Likewise, functional studies confirm the impact of these genes: overexpressing different ATF1/ATF2 alleles in laboratory strains caused dramatic changes in ethyl and isoamyl acetate levels (Liu et al., 2020). Manipulating the medium-chain fatty-acid ester genes gives similar results. In an industrial *S. cerevisiae* strain, partial downregulation of the EEB1 gene (with or without EHT1) sharply lowered ethyl ester production, and in this EEB1-attenuated mutant ATF2 became upregulated (raising acetate esters) (Yang et al., 2022a, Yang et al., 2022b). In sum, allelic differences and expression levels of ATF1, ATF2, EHT1, EEB1 (and related transferase genes) have been directly linked to the observed differences in VOC ester profiles among yeast strains. Notably, esters represented the most diverse VOC category in BV818 and SY, although their concentrations in these samples were relatively low. These esters can significantly influence the final aroma of fruit wines, beverages, and other fermented foods (Jiang et al., 2019; Zhu et al., 2023). In contrast, the BV818 fermentation was characterized by a predominance of acids (3361 μg/L) and alcohols (2281 μg/L), the SY fermentation contained relatively higher levels of other miscellaneous volatiles (1714 μg/L).

Higher alcohols, primarily derived from yeast-mediated amino acid degradation (Wang et al., 2024), represented another critical aroma group influencing the sensory attributes of PFW. FR fermentation resulted in the highest total higher alcohol content, prominently phenylethyl alcohol (19.02 μg/L), responsible for rose-like aromas. Additionally, FR uniquely accumulated substantial 2-methyl-1-propanol (16.43 μg/L), indicating enhanced isoleucine metabolism, aligning with previous studies (Isogai et al., 2022). Minor alcohol differences included the unique presence of n-propanol (2.39 μg/kg) in L2323, while BV818 contained minor (*S*)-2-methyl-1-butanol (1.21 μg/kg), an isoamyl alcohol isomer.

Despite these clear trends, the underlying yeast metabolic mechanisms remain to be elucidated. It would be valuable to measure the expression and activity of key ester-synthesizing enzymes in each strain. For example, assaying alcohol acetyltransferase (AAT) activity in yeast extracts could test whether FR's high ethyl ester levels arise from upregulated AAT function. In yeast, AAT catalyzes the transfer of acetyl-CoA to higher alcohols to form volatile esters; any strain-to-strain differences in these enzymes could explain the observed aroma variation (Ge et al., 2025). It is also known that fermentation conditions influence ATF1 expression – for instance, aeration and unsaturated fatty acids strongly repress ATF1, reducing acetate ester synthesis (Saerens et al., 2010). Thus, future work should also examine how variables like oxygenation, lipid supplementation, or nitrogen status impact ester production in each yeast, as these parameters may modulate gene expression and metabolic flux in the Ehrlich pathway.

Subsequently, to further investigate and present the differences among various PFW samples, an OPLS-DA model was constructed based on the 110 identified VOCs. The cumulative variance contribution of the first two principal components (0.882) exceeded 0.5, indicating that the OPLS-DA model was effective in explaining and predicting the differences among PFW samples (R^2^Y = 0.996, Q^2^ = 0.992). Permutation testing (*n* = 200) validated the model, showing no overfitting (R^2^ = 0.0766, Q^2^ = −0.774) (Li, Huang, et al., 2024). In the OPLS-DA score plot (Fig. 4D), the FR and D254 samples clustered in the fourth quadrant, while SY and L2323 showed a tendency toward grouping and L2323 was clearly separated from the other samples along PC1, indicating that yeast fermentation plays an important role in shaping the flavor profile of PFW. Furthermore, the combination of Fig. 4D and F indicated the distribution of key volatile compounds responsible for discriminating among the PFW samples fermented by different yeast strains. A total of eight VOCs with variable importance in projection (VIP) values greater than 1 were identified as major contributors to sample differentiation (Li, Zhang, et al., 2024). Based on further screening criteria (VIP ≥ 1, *p* < 0.05 or exclusive presence) (Li, Guo, et al., 2024), six potential aroma markers were identified: ethanol, dimethyl ether, 3-sulfo-l-alanine, ethyl nitrate, ethylene glycol dinitrate, and n-propyl acetate. These markers, encompassing alcohols, esters, and heterocyclic compounds, significantly shaped PFW's sensory attributes, imparting diverse aromas ranging from fruity and floral to bitter and caramel-like (Li, Zhang, et al., 2024). In addition, Zhang, Qin, et al. (2023) found that KW fermented by RW contained the highest level of ethyl hexanoate, which has been considered one of the key odor-active compounds in KW. Meanwhile, this information was partly similar to the components of potential flavor markers in diverse fermented fruit wines reported by Lan et al. (2022).

### 3.7 Analysis of odor profiles in different PFW

The contribution of individual VOCs to the overall flavor profile of PFW depends not only on their concentrations but also on their odor thresholds (Xi et al., 2024). To comprehensively evaluate aroma-active compounds, both OAV and ROAV were determined. OAV was calculated as the ratio of the concentration measured by HS-SPME-GC–MS to the compound's odor threshold, whereas ROAV was calculated from the relative VOC content obtained by HS-GC-IMS (Xi et al., 2024). Integrating these approaches enabled the identification of major aroma-impact compounds in PFW fermented with different yeasts.

As summarized in Table 1, a total of 10 VOCs with OAV or ROAV ≥1 were identified as key contributors to the overall aroma. Ethyl 2-methylpentanoate was notable for its high ROAV across all samples, indicating a common impact on PFW aroma profiles. Among strain-specific effects, L2323-fermented PFW was dominated by 3-methyl-1-butanol acetate (OAV = 140.08), which imparted prominent apple and banana notes. In the FR group, ethyl acetate (OAV = 77.53) and ethyl 2-methylpropionate (OAV = 21.04) served as major contributors, providing balsamic, buttery, pungent, and fruity characteristics (Deng et al., 2021). For BV818, ethyl butanoate and 3-methyl-1-butanol acetate were the principal aroma-active compounds, conferring sweet and fruity notes. Most of these aroma-impact compounds were esters, consistent with their dominance among the identified VOCs in PFW. In contrast, SY and D254 fermentations exhibited lower OAV values for major volatiles, suggesting these strains contributed less to the characteristic aroma formation in PFW compared with the other three yeasts.

**Table 1:** The key aromatic compounds of PFW.

Compound Name	Aroma description	Formula	CAS	Thresholds (μg/mL)	OAV/ROAV				
					BV818	L2323	FR	SY	D254
Formic acid	acid, pungent^a^	CH_2_O_2_	64–18-6	0.097	4.17	7.87	8.42	0.98	1.14
Butanoic acid, ethyl ester	anise, apple^a^	C_6_H_12_O_2_	105–54-4	0.0009	30.98	<0.01	<0.01	<0.01	2.05
n-Propyl acetate	celery, floral^a^	C_5_H_10_O_2_	109–60-4	1	0.02	1.76	<0.01	<0.01	<0.01
Hexanoic acid, ethyl ester	apple peel, banana^a^	C_8_H_16_O_2_	123–66-0	0.005	0.53	0.56	1.78	0.06	0.33
3-methyl-1-Butanol acetate	apple, banana^a^	C_7_H_14_O_2_	123–92-2	0.00015	50.35	140.08	<0.01	1.82	19.68
Ethyl Acetate	balsamic, butter^a^	C_4_H_8_O_2_	141–78-6	0.005	1.33	0.00	77.53	0.08	<0.01
ethyl 2-methylpentanoate	anise, fruit^b^	C_8_H_16_O_2_	39,255–32-8	0.000003	100.00	100.00	100.00	100.00	100.00
Ethyl 2-methy lpropionate	apple, floral, fruit^b^	C_6_H_12_O_2_	97–62-1	0.00002	5.84	8.22	21.04	13.91	8.82
Butanal	banana, green^b^	C_4_H_8_O	123–72-8	0.002	1.56	0.56	2.05	0.89	1.76
Nonanal	citrus, cucumber, fat^a^	C_9_H_18_O	124–19-6	0.0011	<0.01	<0.01	<0.01	<0.01	1.66

a: represents the key aromatic compounds selected by OAV based on the results of HS-SPEM-GC–MS.

b: represents the key aromatic compounds selected by ROAV based on the results of HS-GC-IMS.

Note:All the thresholds are derived from https://www.doc88.com/p-2039102934971.html.

## 4 Conclusion

This comprehensive investigation systematically elucidates the pivotal role of yeast strain selection in modulating the physicochemical properties, antioxidant potential, and flavor characteristics of PFW. Strain D254 produced the highest phenolic content and antioxidant activity, whereas FR and L2323 fermentations yielded richer and more diverse esters and higher alcohols, greatly enhancing fruity aroma complexity. Practically, strain choice can be aligned with product targets: D254 for antioxidant- and polyphenol-forward styles (with astringency management during maturation), and FR/L2323 for ester-forward, fruit-driven styles (favoring the cooler end of the recommended temperature window to retain esters). Overall, our findings offer clear, practical guidance for PFW. Odor-activity analysis links chemistry to likely perception; however, no trained sensory panel or consumer acceptance testing was performed. Future work should validate the predicted aroma shifts and quantify preference thresholds for astringency and fruity balance in PFW.

## CRediT authorship contribution statement

**Haibin Yuan:** Writing – original draft, Investigation, Data curation. **Yanlin Li:** Writing – review & editing, Investigation. **Jianhang Tu:** Validation, Resources. **Tianyang Wang:** Validation, Resources. **Kaixian Zhu:** Methodology. **Hongfeng Jia:** Writing – review & editing. **Huachang Wu:** Writing – review & editing, Visualization, Validation, Conceptualization. **Ping Dong:** Writing – review & editing, Writing – original draft, Funding acquisition.

## Declaration of competing interest

The authors declare that they have no known competing financial interests or personal relationships that could have appeared to influence the work reported in this paper.

## Data Availability

Data will be made available on request.

## Appendix A Supplementary data

Supplementary material Image 1
