# Supplementary material for: The impact of S. cerevisiae strains on the aroma profile and physicochemical properties of Phyllanthus emblica L. fruit wine
Source: Food Chem X. 2025 Dec 14;33:103388. doi: 10.1016/j.fochx.2025.103388 (PMC12768916; doi:10.1016/j.fochx.2025.103388)
Supplement: Supplementary file 1 — Supplementary material [file mmc1.docx]

**The impact of *S. cerevisiae* strains on the aroma profile and physicochemical properties of *Phyllanthus emblica L*. fruit wine**

| Table S1. Corresponding substances for electronic nose sensors | | | |
| --- | --- | --- | --- |
| NO | Sensor name | Performance | Reference material |
| 1 | LY2/LG | sensitive to gases with strong oxidation capacity | 2-methyl-3-furan thiol |
| 2 | LY2/G | Sensitive to amine substances | Methylamine |
| 3 | LY2/AA | Sensitive to organic compounds | Valeric aldehyde |
| 4 | LY2/Gh | Selective for aniline | Aniline |
| 5 | LY2/gCTI | Sensitive to amine substances | N-Butylamine |
| 6 | LY2/gCT | Sensitive to alkanes and aromatic components | Propane |
| 7 | T30/1 | Sensitive to polar compounds | Propanol |
| 8 | P10/1 | Sensitive to non-polar compounds | N-octane |
| 9 | P10/2 | Sensitive to alkanes | N-heptane |
| 10 | P40/1 | Sensitive to gases with strong oxidation capacity | Methyl furfuryl disulfide |
| 11 | T70/2 | Sensitive to aromatic compounds | Xylene |
| 12 | PA/2 | Sensitive to organic compounds | Acetaldehyde and amine compounds |
| 13 | P30/1 | Sensitive to organic compounds | Ethanol |
| 14 | P40/2 | Sensitive to gases with strong oxidation capacity | Methyl mercaptan |
| 15 | P30/2 | Sensitive to organic compounds | α -terpineol |
| 16 | T40/2 | Sensitive to gases with strong oxidation capacity | Furfuryl mercaptan |
| 17 | T40/1 | Sensitive to gases with strong oxidation capacity | Dimethyl disulfide |
| 18 | TA/2 | Sensitive to organic compounds | Hexanol |

| **Table. S2.** The relative concentration of VOCs in SV identified by HS-GC-IMS | | | | | | | | | | | |
| --- | --- | --- | --- | --- | --- | --- | --- | --- | --- | --- | --- |
| Compound | CAS | Formula | Actual RI | Theoretical RI | Identification methods | Rt [sec] | BV818 | L2323 | FR | SY | D254 |
| Methyl nonanoate-M | 1731-84-6 | C10H20O2 | 1485 | 1451.6 | IMS，RI | 1487.97 | 6.75 | 6.11 | 5.06 | 5.25 | 6.14 |
| Methyl nonanoate-D | 1731-84-6 | C10H20O2 | 1485 | 1452.8 | IMS，RI | 1491.648 | 2.57 | 1.65 | 0.95 | 1.00 | 1.32 |
| (E)-Ethyl-2-hexenoate-M | 27829-72-7 | C8H14O2 | 1345 | 1321.8 | IMS，RI | 1098.704 | 0.68 | 0.90 | 0.99 | 0.92 | 0.94 |
| (E)-Ethyl-2-hexenoate-D | 27829-72-7 | C8H14O2 | 1345 | 1322.3 | IMS，RI | 1100.078 | 0.09 | 0.11 | 0.12 | 0.10 | 0.12 |
| Ethyl propanoate | 105-37-3 | C5H10O2 | 956 | 958.7 | IMS，RI | 321.756 | 0.68 | 0.65 | 0.86 | 1.03 | 0.72 |
| Butyl acetate-M | 123-86-4 | C6H12O2 | 1069 | 1015.6 | IMS，RI | 367.15 | 0.33 | 0.40 | 0.44 | 0.39 | 0.47 |
| Butyl acetate-D | 123-86-4 | C6H12O2 | 1069 | 1014.8 | IMS，RI | 366.13 | 0.67 | 1.01 | 0.91 | 0.60 | 0.75 |
| Methyl heptanoate-M | 106-73-0 | C8H16O2 | 1287 | 1236.8 | IMS，RI | 829.157 | 21.09 | 17.78 | 13.64 | 11.37 | 12.54 |
| methyl heptanoate-D | 106-73-0 | C8H16O2 | 1287 | 1241.9 | IMS，RI | 845.62 | 4.91 | 5.58 | 6.22 | 6.27 | 6.56 |
| Ethyl 3-ethoxypropanoate | 763-69-9 | C7H14O3 | 1332 | 1292.2 | IMS，RI | 1009.179 | 3.88 | 1.75 | 0.94 | 0.88 | 1.11 |
| Butanoic acid, 3-methylbutyl ester-M | 109-19-3 | C9H18O2 | 1295 | 1279.4 | IMS，RI | 967.478 | 0.14 | 0.12 | 0.13 | 0.12 | 0.13 |
| Butanoic acid, 3-methylbutyl ester-D | 109-19-3 | C9H18O2 | 1295 | 1279.1 | IMS，RI | 966.771 | 0.50 | 0.37 | 0.44 | 0.30 | 0.49 |
| methyl pentanoate-D | 624-24-8 | C6H12O2 | 1077 | 1035.6 | IMS，RI | 391.443 | 3.16 | 2.62 | 1.58 | 1.91 | 1.61 |
| methyl pentanoate-M | 624-24-8 | C6H12O2 | 1077 | 1036.2 | IMS，RI | 392.202 | 1.63 | 1.84 | 2.05 | 2.25 | 2.26 |
| Butanoicac id propyl ester | 105-66-8 | C7H14O2 | 1134 | 1140.7 | IMS，RI | 566.235 | 0.26 | 0.38 | 0.26 | 0.39 | 0.26 |
| ethyl 2-methylpentanoate | 39255-32-8 | C8H16O2 | 1148 | 910.6 | IMS，RI | 294.124 | 0.05 | 0.06 | 0.05 | 0.12 | 0.06 |
| 2-methyl-1-propyl acetate | 110-19-0 | C6H12O2 | 1003 | 987.5 | IMS，RI | 338.304 | 0.12 | 0.21 | 0.14 | 0.16 | 0.13 |
| Ethyl 2-methy lpropionate | 97-62-1 | C6H12O2 | 1006 | 966.9 | IMS，RI | 326.439 | 0.02 | 0.03 | 0.07 | 0.11 | 0.04 |
| 1-Hexanol | 111-27-3 | C6H14O | 1350 | 1360.3 | IMS，RI | 1214.152 | 0.27 | 0.29 | 0.34 | 0.38 | 0.33 |
| 2-Methyl-2-propanol | 75-65-0 | C4H10O | 900 | 967.3 | IMS，RI | 326.705 | 0.08 | 0.13 | 0.19 | 0.27 | 0.14 |
| 5-methyl-2-Furanmethanol | 3857-25-8 | C6H8O2 | 1721 | 995.7 | IMS，RI | 343.044 | 0.03 | 0.02 | 0.02 | 0.02 | 0.02 |
| Damascenone | 23696-85-7 | C13H18O | 1832 | 1361.3 | IMS，RI | 1217.275 | 0.23 | 0.27 | 0.26 | 0.37 | 0.32 |
| cis-Jasmone | 488-10-8 | C11H16O | 1923 | 1393.5 | IMS，RI | 1313.599 | 0.23 | 0.31 | 0.32 | 0.35 | 0.30 |
| (E)--Heptenal | 18829-55-5 | C7H12O | 1316 | 911.4 | IMS，RI | 294.578 | 0.20 | 0.23 | 0.17 | 0.54 | 0.25 |
| Butanal | 123-72-8 | C4H8O | 865 | 845.3 | IMS，RI | 256.556 | 0.50 | 0.23 | 0.64 | 0.70 | 0.76 |
| ( E, E)-2,4-octadienal | 30361-28-5 | C8H12O | 1603 | 1067.4 | IMS，RI | 430.259 | 0.04 | 0.05 | 0.06 | 0.08 | 0.06 |
| 2-Methylbutanoic acid | 600-07-7 | C5H10O2 | 1682 | 838.5 | IMS，RI | 252.665 | 0.03 | 0.02 | 0.03 | 0.05 | 0.04 |
| 2,5-Dimethylfuran | 625-86-5 | C6H8O | 954 | 987.5 | IMS，RI | 338.335 | 0.02 | 0.06 | 0.04 | 0.04 | 0.04 |
| Pyrrolidine-M | 123-75-1 | C4H9N | 1008 | 980.9 | IMS，RI | 334.508 | 0.31 | 0.17 | 0.21 | 0.19 | 0.19 |
| Pyrrolidine-D | 123-75-1 | C4H9N | 1008 | 959.8 | IMS，RI | 322.371 | 0.80 | 0.95 | 1.09 | 1.32 | 1.07 |
| 2-Methylpyrazine-M | 109-08-0 | C5H6N2 | 1267 | 1334.5 | IMS，RI | 1136.755 | 0.22 | 0.15 | 0.13 | 0.11 | 0.11 |
| 2-Methylpyrazine-D | 109-08-0 | C5H6N2 | 1267 | 1188.4 | IMS，RI | 675.08 | 0.10 | 0.11 | 0.13 | 0.15 | 0.10 |
| ethylpyrazine | 13925-00-3 | C6H8N2 | 1332 | 963.9 | IMS，RI | 324.716 | 0.03 | 0.04 | 0.07 | 0.09 | 0.04 |
| N-nitrosomethylethylamine | 10595-95-6 | C3H8N2O | 1369.1 | 823 | IMS，RI | 243.752 | 0.08 | 0.13 | 0.13 | 0.62 | 0.35 |
| Tetrahydrothiophene | 110-01-0 | C4H8S | 1150 | 789.9 | IMS，RI | 224.709 | 0.02 | 0.03 | 0.03 | 0.05 | 0.04 |
| dipropyl disulfide-M | 629-19-6 | C6H14S2 | 1379 | 1098.4 | IMS，RI | 469.633 | 3.93 | 4.73 | 4.84 | 5.44 | 5.19 |
| dipropyl disulfide-D | 629-19-6 | C6H14S2 | 1379 | 1094.1 | IMS，RI | 462.824 | 6.34 | 8.67 | 9.00 | 9.63 | 9.44 |
| hexanenitrile | 628-73-9 | C6H11N | 853 | 839 | IMS，RI | 252.935 | 0.03 | 0.03 | 0.03 | 0.07 | 0.04 |
| 2-Butoxyethanol | 111-76-2 | C3H8N2O | 1404 | 823 | IMS，RI | 243.752 | 0.12 | 0.16 | 0.17 | 0.20 | 0.18 |
| 1-Butanol, 3-methyl-, acetate-M | 123-92-2 | C7H14O2 | 1133 | 1123.8 | IMS，RI | 527.555 | 12.30 | 11.44 | 13.29 | 10.45 | 10.90 |
| 1-Butanol, 3-methyl-, acetate-D | 123-92-2 | C7H14O2 | 1133 | 1126.2 | IMS，RI | 532.944 | 2.63 | 2.72 | 2.95 | 2.78 | 3.13 |
| 1-Propanol | 71-23-8 | C3H8O | 1031 | 1049 | IMS，RI | 407.817 | 0.06 | 0.07 | 0.09 | 0.13 | 0.09 |
| Acetic acid propyl ester | 109-60-4 | C5H10O2 | 965 | 980.1 | IMS，RI | 334.04 | 0.09 | 0.03 | 0.03 | 0.02 | 0.03 |
| 1-Pentanol-M | 71-41-0 | C5H12O | 1247 | 1208 | IMS，RI | 735.31 | 21.43 | 24.86 | 28.02 | 30.08 | 28.13 |
| 1-Pentanol-D | 71-41-0 | C5H12O | 1247 | 1215 | IMS，RI | 757.995 | 2.36 | 2.54 | 2.88 | 2.73 | 3.07 |
| Note: --: not detected | | | | | | | | | | | |

| Table. S3. The relative concentration and retention index (Rt) of VOCs in PFW identified by HS-GC-IMS and HS-SPME-GC–MS | | | | | | | | | | | | | |
| --- | --- | --- | --- | --- | --- | --- | --- | --- | --- | --- | --- | --- | --- |
| NO. | Compound Name | Thresholds | Formula | CAS | Identification methods | Theoretical RI | R.Match | Rt | Concentration (µg/L) | | | | |
|  |  |  |  |  |  |  |  |  | BV818 | L2323 | FR | SY | D254 |
| 1 | Phenethyl alcohol | - | C7H8 | 60-12-8 | MS Rt R.Match | 1903 | 856 | 15.149 | 1.18士0.03c | 2.19士0.05b | 19.02士0.34a | 0.26士0.01d | 2.24士0.22b |
| 2 | 2-Methyl-1-butanol | - | C5H12O | 1565-80-6 | MS Rt R.Match | 1213 | 874 | 6.286 | 1.21士0.06a | - | - | - | - |
| 3 | [(E)-2-pentenol](https://www.chemicalbook.com/Search.aspx?keyword=(E)-2-pentenol" \o "https://www.chemicalbook.com/Search.aspx?keyword=(E)-2-pentenol) | - | C5H10O | 1576-96-1 | MS Rt R.Match | 1321 | 915 | 6.046 | - | 6.09士0.60a | - | - | - |
| 4 | 1-Hepten-4-ol | 0.0892 | C7H14O | 3521-91-3 | MS Rt R.Match | 1585 | 852 | 9.852 | 39.74士2.25a | - | - | - | - |
| 5 | 4-Methyl-1-pentanol |  | C6H14O | 626-89-1 | MS Rt R.Match | 1301 | 864 | 8.422 | 1.89士0.17a | - | - | - | - |
| 6 | Ethanol | 0.82 | C2H6O | 64-17-5 | MS Rt R.Match | 1254 | 825 | 2.289 | 2229.73士30.07b | 162.84士3.97c | 1824.24士543.23a | 161.63士7.09c | 2302.82士103.48b |
| 7 | 1-Propanol | 950 | C3H8O | 71-23-8 | MS Rt R.Match | 1031 | 869 | 5.28 | - | 2.39士0.06a | - | - | - |
| 8 | 1-Pentanol | 8.5056 | C5H12O | 71-41-0 | MS Rt R.Match | 1247 | 884 | 5.651 | 3.90士0.31c | 6.28士0.26b | 9.09士0.65a | 1.52士0.11e | 2.82士0.09d |
| 9 | 2-Methyl-1-propanol | 0.1502 | C4H10O | 78-83-1 | MS Rt R.Match | 1094 | 891 | 5.721 | - | - | 16.43士1.48a | - | - |
| 10 | 1,4-Pentadien-3-ol | 6.5052 | C5H8O | 922-65-6 | MS Rt R.Match | 907.75 | 833 | 11.383 | 1.20士0.01a | - | - | - | - |
| 11 | Leaf alcohol | - | C6H12O | 928-96-1 | MS Rt R.Match | 1380 | 982 | 8.682 | 2.91士0.17c | 3.53士0.20b | 9.87士0.32a | 0.36士0.02e | 1.73士0.12d |
| 12 | 1-Methoxy-2-propanol | 1.9 | C4H10O2 | 107-98-2 | MS Rt R.Match | 1129 | 824 | 2.604 | - | - | - | 0.56士0.04b | 1.57士0.02a |
| 13 | Vinyl ether | 4 | C4H6O | 109-93-3 | MS Rt R.Match | 480 | 865 | 6.856 | - | - | - | 15.94士1.03a | - |
| 14 | Dimethyl ether | - | C2H6O | 115-10-6 | MS Rt R.Match | 478 | 929 | 2.289 | 127.62士11.45d | 460.61士35.83c | 1185.09士80.32b | 1694.48士122.16a | 1727.53士91.04a |
| 15 | Trimethylacetic anhydride | - | C10H18O3 | 1538-75-6 | MS Rt R.Match | 1053 | 904 | 9.747 | - | 2.85士0.28b | 7.47士0.49a | - | - |
| 16 | Acrylic anhydride | - | C6H6O3 | 2051-76-5 | MS Rt R.Match | 1182 | 880 | 6.696 | - | - | - | - | 64.24士2.69a |
| 17 | Valeric anhydride | - | C10H18O3 | 2082-59-9 | MS Rt R.Match | 1283.1 | 893 | 5.27 | 19.14士1.13a | 21.65士1.92a | 9.17士0.85b | 3.25士0.28c | 3.92士0.33c |
| 18 | Undecan-4-olide | - | C11H20O2 | 104-67-6 | MS Rt R.Match | 2266 | 863 | 6.066 | - | - | - | - | 12.61士0.52a |
| 19 | Nonanal | 0.03 | C9H18O | 124-19-6 | MS Rt R.Match | 1389 | 958 | 17.19 | - | - | - | - | 1.83士0.15a |
| 20 | 2,2-dimethylvaleraldehyde | 0.0011 | C7H14O | 14250-88-5 | MS Rt R.Match | 907.75 | 898 | 14.144 | 1.07士0.03a | - | - | - | - |
| 21 | 2-Butenal | 0.041 | C4H6O | 123-73-9 | MS Rt R.Match | 1036 | 885 | 3.195 | 84.48士6.74a | - | - | - | - |
| 22 | 2-Propynal | - | C3H2O | 624-67-9 | MS Rt R.Match | 901 | 972 | 2.609 | - | - | - | - | 4.33士0.41a |
| 23 | Pivaldehyde | - | C5H10O | 630-19-3 | MS Rt R.Match | 809 | 889 | 11.583 | - | - | - | 0.22士0.00a | - |
| 24 | Ethyl (z)-4-decenoate | 0.078 | C12H22O2 | 7367-84-2 | MS Rt R.Match | 1361 | 832 | 27.284 | - | 2.59士0.15a | - | - | - |
| 25 | Aminomethanesulfonic acid | - | CH5NO3S | 13881-91-9 | MS Rt R.Match | 1920.6 | 895 | 2.299 | - | - | 102.03士9.79a | - | - |
| 26 | Oxalic acid | - | C2H2O4 | 144-62-7 | MS Rt R.Match | 748 | 849 | 2.659 | - | - | 14.31士0.65a | - | - |
| 27 | 2-Ethyl-heptanoic acid | - | C9H18O2 | 3274-29-1 | MS Rt R.Match | 1538.69 | 904 | 19.841 | - | 4.44士0.35b | 12.66士0.42a | 0.29士0.02c | 3.86士0.20b |
| 28 | L-Cysteuc acid | - | C3H7NO5S | 498-40-8 | MS Rt R.Match | 2098.47 | 931 | 2.059 | 2956.92士158.27a | - | - | - | - |
| 29 | Ethoxyacetic acid | - | C4H8O3 | 627-03-2 | MS Rt R.Match | 1081.4 | 808 | 5.33 | - | - | 11.88士0.68a | - | - |
| 30 | Formic acid | - | CH2O2 | 64-18-6 | MS Rt R.Match | 1535 | 898 | 2.659 | 404.37士8.70b | 763.52士56.61a | 817.21士23.56a | 95.17士4.40c | 110.84士14.69c |
| 31 | Acetic acid | 0.097 | C2H4O2 | 64-19-7 | MS Rt R.Match | 1443 | 961 | 5.951 | - | - | 9.18士0.56a | - | - |
| 32 | Isopropylbarbituric acid | 99 | C7H10N2O3 | 7391-69-7 | MS Rt R.Match | 2264 | 965 | 11.658 | - | 2.25士0.12a | - | - | - |
| 33 | 3-Heptanone | - | C7H14O | 106-35-4 | MS Rt R.Match | 1154 | 915 | 9.407 | 69.56士4.50a | - | - | - | 1.88士0.13b |
| 34 | 4-Methyl-5-nonanone | 0.08 | C10H20O | 35900-26-6 | MS Rt R.Match | 1529 | 897 | 5.015 | - | 3.77士0.16a | - | - | - |
| 35 | 2-Octen-4-one | 0.075 | C8H14O | 4643-27-0 | MS Rt R.Match | 1309.2 | 915 | 11.733 | 1.33士0.05d | 2.82士0.28b | 5.98士0.37a | 0.24士0.02e | 2.25士0.09c |
| 36 | 5-Nonanone | - | C9H18O | 502-56-7 | MS Rt R.Match | 1334.1 | 837 | 15.905 | 1.45士0.10d | 2.69士0.16b | 6.44士0.31a | 0.21士0.01e | 2.20士0.10c |
| 37 | [Artemesia](https://www.chemicalbook.com/Search.aspx?keyword=Artemesia) | - | C10H16O | 546-49-6 | MS Rt R.Match | 1348 | 843 | 11.763 | - | - | 6.35士0.39a | 0.22士0.01b | 1.80士0.11b |
| 38 | 4-Octanone | 0.000026 | C8H16O | 589-63-9 | MS Rt R.Match | 1197 | 959 | 12.023 | 2.41士0.07b | 2.31士0.12b | 5.44士0.49a | 2.59士0.18b | - |
| 39 | 5-Methyl-4-octanone | - | C9H18O | 6175-51-5 | MS Rt R.Match | 1361.8 | 804 | 12.483 | - | - | - | - | 2.83士0.25a |
| 40 | Styrene | 0.041 | C8H8 | 100-42-5 | MS Rt R.Match | 1263 | 814 | 9.887 | - | 4.21士0.39c | 12.52士0.81b | 0.39士0.03d | 17.26士0.32a |
| 41 | 2-Pentene | - | C5H10 | 109-68-2 | MS Rt R.Match | 1144 | 940 | 5.851 | - | - | 7.76士0.29a | - | - |
| 42 | 6-Methyl-1-heptene | 0.065 | C8H16 | 5026-76-6 | MS Rt R.Match | 746.2 | 887 | 8.812 | - | - | - | - | 2.14士0.11a |
| 43 | Cycloheptatriene | - | C7H8 | 544-25-2 | MS Rt R.Match | 1089 | 844 | 17.49 | 1.41士0.08a | - | - | - | - |
| 44 | 2-Methyl-1-butene | - | C5H10 | 563-46-2 | MS Rt R.Match | 534 | 858 | 5.781 | - | 134.15士11.92a | - | - | - |
| 45 | 1,4-Pentadiene | - | C5H8 | 591-93-5 | MS Rt R.Match | 646 | 889 | 6.886 | - | - | - | - | 2.39士0.23a |
| 46 | Phenethyl acetate | - | C10H12O2 | 103-45-7 | MS Rt R.Match | 1833 | 892 | 22.667 | - | - | 5.82士0.16a | 0.23士0.01b | - |
| 47 | Ethyl butyrate | - | C_6_H_12_O_2_ | 105-54-4 | MS Rt R.Match | 1030 | 839 | 7.131 | 27.89士2.58a | - | - | - | 1.85士0.12b |
| 48 | Ethyl caprylate | 0.24959 | C10H20O2 | 106-32-1 | MS Rt R.Match | 1427 | 933 | 19.561 | 2.22士0.19d | 3.57士0.24c | 10.41士0.36a | 0.70士0.03e | 4.83士0.56b |
| 49 | Propyl acetate | 0.0009 | C5H10O2 | 109-60-4 | MS Rt R.Match | 965 | 841 | 2.975 | 22.15士1.63b | 1757.09士176.14a | - | 0.57士0.02d | 2.98士0.11c |
| 50 | Ethyl caprate | 0.0193 | C12H24O2 | 110-38-3 | MS Rt R.Match | 1618 | 969 | 27.459 | 2.34士0.19b | 2.22士0.06bc | 6.12士0.22a | 0.25士0.02d | 1.85士0.19c |
| 51 | Caprylic acid methyl ester | 1 | C9H18O2 | 111-11-5 | MS Rt R.Match | 1349 | 815 | 17.835 | 0.93士0.08c | 2.58士0.16b | 7.08士0.29a | 0.29士0.02c | - |
| 52 | Ethyl nonanoate | 2 | C11H22O2 | 123-29-5 | MS Rt R.Match | 1528 | 945 | 19.841 | 1.31士0.08a | 2.02士0.17a | - | - | - |
| 53 | Ethyl Hexanoate | 0.2 | C8H16O2 | 123-66-0 | MS Rt R.Match | 1232 | 916 | 7.966 | 2.67士0.18b | 2.79士0.13b | 8.91士0.40a | 0.30士0.02d | 1.67士0.17c |
| 54 | Isoamyl acetate | 0.377 | C7H14O2 | 123-92-2 | MS Rt R.Match | 1232 | 917 | 8.787 | 7.55士0.43b | 21.01士1.91a | - | 0.27士0.01d | 2.95士0.10c |
| 55 | Ethyl 2-isocyanatopropionate | 0.005 | C6H9NO3 | 13794-28-0 | MS Rt R.Match | 1525 | 955 | 5.951 | - | 80.46士1.09a | - | - | - |
| 56 | Ethyl Acetate | 0.00015 | C4H8O2 | 141-78-6 | MS Rt R.Match | 884 | 836 | 7.451 | 6.67士0.48b | - | 387.64士19.91a | 0.42士0.04b | - |
| 57 | Methyl thiolacetate | - | C3H6OS | 1534-08-3 | MS Rt R.Match | 1065 | 930 | 22.792 | 1.85士0.13a | - | - | - | - |
| 58 | [2-Methylpentanoic acid methyl ester](https://www.chemicalbook.com/Search.aspx?keyword=2-Methylpentanoic acid methyl ester" \o "https://www.chemicalbook.com/Search.aspx?keyword=2-Methylpentanoic acid methyl ester) | 0.005 | C7H14O2 | 2177-77-7 | MS Rt R.Match | 877 | 895 | 20.121 | - | - | - | 0.41士0.02a | - |
| 59 | 3-Hexenyl acetate | - | C8H14O2 | 3681-71-8 | MS Rt R.Match | 1319 | 842 | 13.429 | 1.07士0.05d | 2.38士0.24b | 7.91士0.08a | 0.34士0.00e | 2.01士0.09c |
| 60 | Ethyl valerate | - | C7H14O2 | 539-82-2 | MS Rt R.Match | 1132 | 924 | 10.087 | - | - | - | - | 2.24士0.07a |
| 61 | Propyl isovalerate | 0.031 | C8H16O2 | 557-00-6 | MS Rt R.Match | 1140 | 856 | 16.5 | 1.66士0.08a | - | - | - | - |
| 62 | Ethyl 3-methylvalerate | 0.0058 | C8H16O2 | 5870-68-8 | MS Rt R.Match | 957 | 843 | 10.367 | 5.35士0.33a | 4.42士0.23b | - | 0.30士0.01c | - |
| 63 | Methyl nitrate | 0.0087 | CH3NO3 | 598-58-3 | MS Rt R.Match | 548 | 881 | 2.549 | 35.47士2.29d | 385.83士19.94c | 5840.23士289.95b | 12.21士1.03e | 11851.23士68.60a |
| 64 | [1-pentylpropanoate](https://www.chemicalbook.com/Search.aspx?keyword=1-pentylpropanoate) | 0.000008 | C8H16O2 | 624-54-4 | MS Rt R.Match | 1244 | 814 | 29.31 | 1.07士0.08a | - | - | - | - |
| 65 | Chloromethyl acetate | - | C3H5ClO2 | 625-56-9 | MS Rt R.Match | 1164 | 854 | 8.952 | 1.21士0.08d | 16.94士1.16a | 13.57士1.05b | 0.60士0.05e | 8.00士0.68c |
| 66 | [Ethyl undecylate](https://www.chemicalbook.com/Search.aspx?keyword=Ethyl undecylate" \o "https://www.chemicalbook.com/Search.aspx?keyword=Ethyl undecylate) | - | C13H26O2 | 627-90-7 | MS Rt R.Match | 1733 | 865 | 20.556 | 1.33士0.06a | - | - | - | - |
| 67 | EGDN | - | C2H4N2O6 | 628-96-6 | MS Rt R.Match | 1032.05 | 800 | 2.309 | 21.27士1.41d | 107.68士5.46c | 1149.29士58.72a | 111.29士2.39c | 383.74士35.29b |
| 68 | [1-pentylformate](https://www.chemicalbook.com/Search.aspx?keyword=1-pentylformate) | - | C6H12O2 | 638-49-3 | MS Rt R.Match | 1258 | 852 | 9.492 | - | - | - | 0.27士0.01a | - |
| 69 | 1,2-Propanediol, dinitrate | - | C3H6N2O6 | 6423-43-4 | MS Rt R.Match | 1004 | 841 | 2.244 | 109.22士2.40d | 6969.94士446.91b | 16538.88士445.09a | 199.40士9.88c | 6428.39士616.02b |
| 70 | [Pentanoic acid, 2-hydroxy-, ethyl ester](https://www.chemicalbook.com/Search.aspx?keyword=Pentanoic acid, 2-hydroxy-, ethyl ester" \o "https://www.chemicalbook.com/Search.aspx?keyword=Pentanoic acid, 2-hydroxy-, ethyl ester) | - | C7H14O3 | 6938-26-7 | MS Rt R.Match | 1381 | 946 | 3.43 | 16.07士1.18a | - | - | - | - |
| 71 | Ethyl trans-4-decenoate | - | C12H22O2 | 76649-16-6 | MS Rt R.Match | 1382 | 931 | 27.309 | 2.34士0.06c | 2.56士0.13bc | 8.44士0.51a | 0.54士0.04d | 3.13士0.14b |
| 72 | Methyl nonanoate-M | 0.04 | C10H20O2 | 1731-84-6 | IMS，RI | 1485 | Actual RI | 1487.97 | 6.75 | 6.11 | 5.06 | 5.25 | 6.14 |
| 73 |  |  |  |  |  |  | 1451.6 |  |  |  |  |  |  |
| 74 | Methyl nonanoate-D | 0.04 | C10H20O2 | 1731-84-6 | IMS，RI | 1485 | 1452.8 | 1491.648 | 2.57 | 1.65 | 0.95 | 1.00 | 1.32 |
| 75 | (E)-Ethyl-2-hexenoate-M | 0.14 | C8H14O2 | 27829-72-7 | IMS，RI | 1345 | 1321.8 | 1098.704 | 0.68 | 0.90 | 0.99 | 0.92 | 0.94 |
| 76 | (E)-Ethyl-2-hexenoate-D | 0.14 | C8H14O2 | 27829-72-7 | IMS，RI | 1345 | 1322.3 | 1100.078 | 0.09 | 0.11 | 0.12 | 0.10 | 0.12 |
| 77 | Ethyl propanoate | 0.01 | C5H10O2 | 105-37-3 | IMS，RI | 956 | 958.7 | 321.756 | 0.68 | 0.65 | 0.86 | 1.03 | 0.72 |
| 78 | Butyl acetate-M | 0.058 | C6H12O2 | 123-86-4 | IMS，RI | 1069 | 1015.6 | 367.15 | 0.33 | 0.40 | 0.44 | 0.39 | 0.47 |
| 79 | Butyl acetate-D | 0.058 | C6H12O2 | 123-86-4 | IMS，RI | 1069 | 1014.8 | 366.13 | 0.67 | 1.01 | 0.91 | 0.60 | 0.75 |
| 80 | Methyl heptanoate-M | 0.004 | C8H16O2 | 106-73-0 | IMS，RI | 1287 | 1236.8 | 829.157 | 21.09 | 17.78 | 13.64 | 11.37 | 12.54 |
| 81 | methyl heptanoate-D | 0.004 | C8H16O2 | 106-73-0 | IMS，RI | 1287 | 1241.9 | 845.62 | 4.91 | 5.58 | 6.22 | 6.27 | 6.56 |
| 82 | Ethyl 3-ethoxypropanoate | 0.0011 | C7H14O3 | 763-69-9 | IMS，RI | 1332 | 1292.2 | 1009.179 | 3.88 | 1.75 | 0.94 | 0.88 | 1.11 |
| 83 | methyl pentanoate-D | 0.02 | C6H12O2 | 624-24-8 | IMS，RI | 1077 | 1035.6 | 391.443 | 3.16 | 2.62 | 1.58 | 1.91 | 1.61 |
| 84 | methyl pentanoate-M | 0.02 | C6H12O2 | 624-24-8 | IMS，RI | 1077 | 1036.2 | 392.202 | 1.63 | 1.84 | 2.05 | 2.25 | 2.26 |
| 85 | Butanoicac id propyl ester | 0.018 | C7H14O2 | 105-66-8 | IMS，RI | 1134 | 1140.7 | 566.235 | 0.26 | 0.38 | 0.26 | 0.39 | 0.26 |
| 86 | ethyl 2-methylpentanoate | 0.000003 | C8H16O2 | 39255-32-8 | IMS，RI | 1148 | 910.6 | 294.124 | 0.05 | 0.06 | 0.05 | 0.12 | 0.06 |
| 87 | 2-methyl-1-propyl acetate | 0.025 | C6H12O2 | 110-19-0 | IMS，RI | 1003 | 987.5 | 338.304 | 0.12 | 0.21 | 0.14 | 0.16 | 0.13 |
| 88 | Ethyl 2-methy lpropionate | 0.00002 | C6H12O2 | 97-62-1 | IMS，RI | 1006 | 966.9 | 326.439 | 0.02 | 0.03 | 0.07 | 0.11 | 0.04 |
| 89 | 1-Hexanol | 0.0056 | C6H14O | 111-27-3 | IMS，RI | 1350 | 1360.3 | 1214.152 | 0.27 | 0.29 | 0.34 | 0.38 | 0.33 |
| 90 | 2-Methyl-2-propanol | 8.2 | C4H10O | 75-65-0 | IMS，RI | 900 | 967.3 | 326.705 | 0.08 | 0.13 | 0.19 | 0.27 | 0.14 |
| 91 | 5-methyl-2-Furanmethanol | - | C6H8O2 | 3857-25-8 | IMS，RI | 1721 | 995.7 | 343.044 | 0.03 | 0.02 | 0.02 | 0.02 | 0.02 |
| 92 | Damascenone | - | C13H18O | 23696-85-7 | IMS，RI | 1832 | 1361.3 | 1217.275 | 0.23 | 0.27 | 0.26 | 0.37 | 0.32 |
| 93 | cis-Jasmone | - | C11H16O | 488-10-8 | IMS，RI | 1923 | 1393.5 | 1313.599 | 0.23 | 0.31 | 0.32 | 0.35 | 0.30 |
| 94 | (E)--Heptenal | 0.04 | C7H12O | 18829-55-5 | IMS，RI | 1316 | 911.4 | 294.578 | 0.20 | 0.23 | 0.17 | 0.54 | 0.25 |
| 95 | Butanal | 0.002 | C4H8O | 123-72-8 | IMS，RI | 865 | 845.3 | 256.556 | 0.50 | 0.23 | 0.64 | 0.70 | 0.76 |
| 96 | ( E, E)-2,4-octadienal | 0.01 | C8H12O | 30361-28-5 | IMS，RI | 1603 | 1067.4 | 430.259 | 0.04 | 0.05 | 0.06 | 0.08 | 0.06 |
| 97 | 2-Methylbutanoic acid | - | C5H10O2 | 600-07-7 | IMS，RI | 1682 | 838.5 | 252.665 | 0.03 | 0.02 | 0.03 | 0.05 | 0.04 |
| 98 | 2,5-Dimethylfuran | 100 | C6H8O | 625-86-5 | IMS，RI | 954 | 987.5 | 338.335 | 0.02 | 0.06 | 0.04 | 0.04 | 0.04 |
| 99 | Pyrrolidine-M | 20.2 | C4H9N | 123-75-1 | IMS，RI | 1008 | 980.9 | 334.508 | 0.31 | 0.17 | 0.21 | 0.19 | 0.19 |
| 100 | Pyrrolidine-D | 20.2 | C4H9N | 123-75-1 | IMS，RI | 1008 | 959.8 | 322.371 | 0.80 | 0.95 | 1.09 | 1.32 | 1.07 |
| 101 | 2-Methylpyrazine-M | 30 | C5H6N2 | 109-08-0 | IMS，RI | 1267 | 1334.5 | 1136.755 | 0.22 | 0.15 | 0.13 | 0.11 | 0.11 |
| 102 | 2-Methylpyrazine-D | 30 | C5H6N2 | 109-08-0 | IMS，RI | 1267 | 1188.4 | 675.08 | 0.10 | 0.11 | 0.13 | 0.15 | 0.10 |
| 103 | ethylpyrazine | 4 | C6H8N2 | 13925-00-3 | IMS，RI | 1332 | 963.9 | 324.716 | 0.03 | 0.04 | 0.07 | 0.09 | 0.04 |
| 104 | N-nitrosomethylethylamine | - | C3H8N2O | 10595-95-6 | IMS，RI | 1369.1 | 823 | 243.752 | 0.08 | 0.13 | 0.13 | 0.62 | 0.35 |
| 105 | Tetrahydrothiophene | 0.0018 | C4H8S | 110-01-0 | IMS，RI | 1150 | 789.9 | 224.709 | 0.02 | 0.03 | 0.03 | 0.05 | 0.04 |
| 106 | dipropyl disulfide-M | 0.13 | C6H14S2 | 629-19-6 | IMS，RI | 1379 | 1098.4 | 469.633 | 3.934834819 | 4.727969188 | 4.843808488 | 5.444863059 | 5.186863514 |
| 107 | dipropyl disulfide-D | 0.13 | C6H14S2 | 629-19-6 | IMS，RI | 1379 | 1094.1 | 462.824 | 6.338761708 | 8.665154286 | 8.999267305 | 9.628622425 | 9.439757185 |
| 108 | hexanenitrile | 0.0032 | C6H11N | 628-73-9 | IMS，RI | 853 | 839 | 252.935 | 0.02621651 | 0.03469614 | 0.031755775 | 0.074679281 | 0.041566673 |
| 109 | 2-Butoxyethanol | 0.88 | C3H8N2O | 111-76-2 | IMS，RI | 1404 | 823 | 243.752 | 0.117941541 | 0.156339331 | 0.167616014 | 0.195112348 | 0.182219074 |
| 110 | Acetic acid propyl ester | 2 | C5H10O2 | 109-60-4 | IMS，RI | 965 | 980.1 | 334.04 | 0.089622208 | 0.029181668 | 0.032663754 | 0.020994725 | 0.025127779 |
| Note: --: not detected | | | | | | | | | | | | | |
